# Targeted lipidomics dataset of central nervous system and plasma from mice with experimental autoimmune encephalomyelitis

**DOI:** 10.1016/j.dib.2025.111948

**Published:** 2025-08-05

**Authors:** Jörn Lötsch, Irmgard Tegder, Natasja de Bruin, Dominique Thomas, Gerd Geisslinger

**Affiliations:** a Goethe University, Institute of Clinical Pharmacology, Faculty of Medicine, Theodor-Stern-Kai 7, 60590 Frankfurt am Main, Germany; b University of Helsinki, Faculty of Medicine, Haartmaninkatu 8, P.O. Box 63, 00014, Finland; c Fraunhofer Institute for Translational Medicine and Pharmacology (ITMP), Theodor-Stern-Kai 7, 60596 Frankfurt am Main, Germany; d Fraunhofer Cluster of Excellence Immune Mediated Diseases (CIMD), Theodor-Stern-Kai 7, 60596 Frankfurt am Main, Germany; e Present affiliation: benfovir AG, Gräfenhäuser Str. 26, 64293 Darmstadt, Germany

**Keywords:** Preclinical pharmacology, Data science, Machine learning, Lipidomics, Multiple sclerosis, Mouse model

## Abstract

This dataset was generated from a preclinical study that used the relapsing-remitting experimental autoimmune encephalomyelitis (EAE) model in SJL/J mice to examine lipid signalling in neuroinflammation. The study examined how the reference compounds FTY720 (fingolimod, 0.5 mg/kg/day) affected the autotaxin/lysophosphatidic acid (ATX/LPA) axis and related lipid mediators. The mice were divided into three groups: control (no EAE), EAE, and EAE plus fingolimod. Tissue samples were collected from the plasma, cerebellum, hippocampus, and prefrontal cortex, resulting in 26 biological samples. Targeted lipidomics was performed using liquid chromatography-tandem mass spectrometry (LC-MS/MS) to quantify 62 lipid species, including lysophosphatidic acids, ceramides, sphingoid bases, and endocannabinoids. The dataset is provided in both raw and imputed formats, along with comprehensive sample-level metadata.

The data are organized into three comma-separated values (CSV) files: (1) the original quantitative lipidomics data matrix with missing values, (2) a log₁₀-transformed imputed dataset with missing values addressed using random forest imputation, and (3) a metadata file detailing the characteristics of the samples, group assignments, and tissue type. Standardized variable naming and detailed metadata facilitate cross-referencing and integration with other datasets.

This resource enables comparative analyses of lipid profiles across tissues and treatment groups. It supports statistical and machine learning applications and enables the evaluation of data augmentation strategies, including statistical and generative AI approaches. This dataset can be reused in studies of neuroinflammation, lipid signalling, and biomarker discovery, as well as in the development of methods for computational biology and omics data analysis.

Specifications Table

**Table:** 

Subject	Health Sciences, Medical Sciences & Pharmacology
Specific subject area	Targeted lipidomics profiling in mouse neuroinflammation and experimental autoimmune encephalomyelitis
Type of data	Table, Figure Raw, Processed, Imputed, Transformed
Data collection	Lipidomics data were collected from SJL/J mice using the relapsing-remitting experimental autoimmune encephalomyelitis (EAE) model. Plasma, cerebellum, hippocampus, and prefrontal cortex samples were analyzed. Quantification of 62 lipid species was performed by targeted liquid chromatography-tandem mass spectrometry (LC-MS/MS) using a QTrap 5500 (AB Sciex, Germany). Lipids were extracted via liquid-liquid extraction, separated on a C18 reversed-phase column, and detected in positive or negative electrospray ionization mode. Internal standards and calibration curves ensure accuracy. Data were processed and normalized using validated software, with missing values imputed by random forest-based methods. Samples were assigned to control, EAE, or EAE plus fingolimod treatment groups.
Data source location	• Goethe University, Institute of Clinical Pharmacology, Faculty of Medicine, Theodor-Stern-Kai 7, 60,590 Frankfurt am Main, Germany • Fraunhofer Institute for Translational Medicine and Pharmacology (ITMP), Theodor-Stern-Kai 7, 60,596 Frankfurt am Main, Germany
Data accessibility	Targeted lipidomics dataset of central nervous system and plasma from mice with experimental autoimmune encephalomyelitis Repository name: Mendeley Data Data identification number: DOI: 10.17632/m2p6rr9v36.1 Direct URL to data: https://data.mendeley.com/datasets/m2p6rr9v36/1 Instructions for accessing these data: Dataset name in Mendeley data: “Targeted lipidomics dataset of central nervous system and plasma from mice with experimental autoimmune encephalomyelitis”
Related research article	None

## 1 Value of the Data

• **Coverage of lipid signaling in neuroinflammation:**
  This dataset provides quantitative lipidomics profiles of 62 lipid variables of four lipid species, including lysophosphatidic acids, ceramides, sphingoid bases, and endocannabinoids, across four central and peripheral nervous system tissues in a well-characterized mouse model of neuroinflammation. This enables detailed exploration of lipid signaling pathways relevant to multiple sclerosis and related neuroimmune disorders.
• **Enables cross-tissue and treatment group comparisons:**
  Samples from plasma, cerebellum, hippocampus, and prefrontal cortex, across three experimental groups (control, EAE, EAE plus fingolimod), allow robust comparative analyses of tissue-specific and systemic lipid alterations in response to neuroinflammatory processes and therapeutic intervention.
• **Facilitates data re-use and integration:**
  The dataset is provided in both raw and imputed formats, with standardized variable names and comprehensive metadata. This structure supports immediate reuse by other researchers for statistical, multivariate, or machine learning workflows, and enables integration with other omics or preclinical datasets.
• **Statistical power and suitability for data augmentation:**
  Expert review confirms that the sample size and structure provide sufficient statistical power for group comparisons and multivariate modeling. This qualifies the dataset as a benchmark for assessing statistical and generative AI-based data augmentation strategies, supporting the development and validation of new computational methods for omics data.
• **Supports translational and preclinical research:**
  Leveraging the widely used EAE model, which recapitulates key features of multiple sclerosis, makes these data directly relevant for translational studies aiming to identify lipid biomarkers, therapeutic targets, or mechanistic insights into neuroimmune modulation

## 2 Background

This dataset was collected during preclinical investigation [1,2] that employed the relapsing-remitting experimental autoimmune encephalomyelitis (EAE) model [3] in SJL/J mice. The study assessed the effects of the reference compound FTY720 (fingolimod, 0.5 mg/kg/day) on the autotaxin/lysophosphatidic acid (ATX/LPA) axis and related lipid signaling molecules in neuroinflammation. Fingolimod [4] is a sphingosine-1-phosphate receptor modulator approved for treating relapsing-remitting multiple sclerosis (RRMS). It modulates lysophospholipid signaling [5].

Lysophosphatidic acids (LPAs) are bioactive lipids that act through specific G-protein-coupled receptors (LPAR1–5 and LPAR6). These receptors are differentially expressed by immune cells and respond to distinct LPA species. The ATX/LPA pathway is involved in immune cell migration, neurogenesis, and blood-brain barrier function. However, its role in MS and animal models is not fully understood. To address this knowledge gap, fingolimod and dimethylfumarate (DMF) were used in a placebo-controlled preclinical study. Targeted lipidomics was performed on plasma and brain tissues to quantify sphingolipids, endocannabinoids, and related molecules. The aim was to characterize the molecular changes associated with these treatments in neuroinflammatory conditions.

## 3 Data Description

The dataset provides quantitative lipidomics measurements from 26 SJL/J mouse samples, each collected from one of four tissue types: plasma, cerebellum, hippocampus, and prefrontal cortex. For each sample, 62 lipid variables of four different species were quantified, covering major lipid classes such as endocannabinoids, ceramides, sphingoid bases, and lysophosphatidic acids (Table 1). Samples are distributed across three different treatment groups, i.e. (i) no experimental autoimmune encephalomyelitis (EAE) = control (*n* = 10), (ii) EAE (*n* = 8), and (iii) EAE plus fingolimod (*n* = 8), and are accompanied by 10 metadata variables per sample, including group and treatment assignment.

**Table 1:** Column variable names, tissue codes and lipid classes for quantitative lipidomics variables used in this study. The variables in this table are quantitative measurements of individual lipid species across four tissue types—cerebellum (CEREB), hippocampus (HPC), prefrontal cortex (PFC), and plasma (PL). Each variable name corresponds exactly to a column in the dataset and consists of a tissue prefix followed by the lipid annotation; for example, “PFC_AEA” denotes anandamide (AEA) abundance measured in prefrontal cortex, and “PL_Cer d18:1/16:0” indicates ceramide (Cer) species with a d18:1 sphingoid base and a 16:0 fatty acid measured in plasma. Lipid species names conform to standard lipidomics nomenclature (e.g., “Cer d18:1/16:0” for ceramide species), and specific sphingoid bases and lysophosphatidic acid (LPA) species are indicated by detailed abbreviations such as “SPH d18:0” (sphinganine), “SPH d18:1” (sphingosine), and “LPA18:1” (lysophosphatidic acid with an 18:1 acyl chain). The suffix “S1P” designates sphingosine-1-phosphate, and all abbreviations match their full chemical names as listed in the analyte column. All values represent quantitative measurements suitable for comparative and multivariate analyses. The broader lipid class and analytical method for each variable are indicated.

Variable	Tissue(s)	Lipid Class	Analyte	Method
CEREB_AG, PFC_AG, PL_AG	CEREB, PFC, PL	Endocannabinoids	1-AG, 2-AG	Endocannabinoids targeted
CEREB_1-AG, PFC_1-AG, PL_1-AG	CEREB, PFC, PL	Endocannabinoids	1-AG	Endocannabinoids targeted
CEREB_2-AG, PFC_2-AG, PL_2-AG	CEREB, PFC, PL	Endocannabinoids	2-AG	Endocannabinoids targeted
CEREB_AEA, PFC_AEA, PL_AEA	CEREB, PFC, PL	Endocannabinoids	AEA	Endocannabinoids targeted
CEREB_OEA, PFC_OEA, PL_OEA	CEREB, PFC, PL	Endocannabinoids	OEA	Endocannabinoids targeted
CEREB_PEA, PFC_PEA, PL_PEA	CEREB, PFC, PL	Endocannabinoids	PEA	Endocannabinoids targeted
CEREB_Cer d18:1/16:0, HPC_Cer d18:1/16:0, PFC_Cer d18:1/16:0, PL_Cer d18:1/16:0	CEREB, HPC, PFC, PL	Ceramides	Cer 18:1;O2/16:0	Ceramide targeted/screening
CEREB_Cer d18:1/18:0, HPC_Cer d18:1/18:0, PFC_Cer d18:1/18:0	CEREB, HPC, PFC	Ceramides	Cer 18:1;O2/18:0	Ceramide targeted/screening
CEREB_Cer d18:1/20:0, HPC_Cer d18:1/20:0, PFC_Cer d18:1/20:0, PL_Cer d18:1/20:0	CEREB, HPC, PFC, PL	Ceramides	Cer 18:1;O2/20:0	Ceramide targeted/screening
PL_Cer d18:1/24:0	PL	Ceramides	Cer 18:1;O2/24:0	Ceramide targeted/screening
PL_Cer d18:1/24:1	PL	Ceramides	Cer 18:1;O2/24:1	Ceramide targeted/screening
CEREB_Cer d18:0/18:0, HPC_Cer d18:0/18:0, PFC_Cer d18:0/18:0, PL_Cer d18:0/18:0	CEREB, HPC, PFC, PL	Sphingoid bases	SPBP 18:0;OH (Sphinganine)	Sphingoid bases targeted
PL_Cer d18:0/16:0	PL	Sphingoid bases	SPBP 16:1;O2 (Sphinganine)	Sphingoid bases targeted
PL_Cer d18:0/24:0	PL	Sphingoid bases	Sphinganine 24:0 (likely)	Sphingoid bases targeted
PL_Cer d18:0/24:1	PL	Sphingoid bases	Sphinganine 24:1 (likely)	Sphingoid bases targeted
CEREB_SPH d18:0, HPC_SPH d18:0, PFC_SPH d18:0, PL_SPH d18:0	CEREB, HPC, PFC, PL	Sphingoid bases	Sphinganine (general)	Sphingoid bases targeted
CEREB_SPH d18:1, HPC_SPH d18:1, PFC_SPH d18:1, PL_SPH d18:1	CEREB, HPC, PFC, PL	Sphingoid bases	SPBP 18:2;O2 (Sphingosine)	Sphingoid bases targeted

Abbreviations: CEREB, cerebellum; HPC, hippocampus; PFC, prefrontal cortex; PL, plasma; Cer, ceramide; LPA, lysophosphatidic acid; SPH, sphingoid base; S1P, sphingosine-1-phosphate; AEA, anandamide; OEA, oleoylethanolamide; PEA, palmitoylethanolamide; AG, monoacylglycerol (1-AG or 2-AG).

All data are organized in three comma-separated values (CSV) files, aligned by sample order. The data set includes the three files:

1. mouse_lipidomics_data_raw.csv: the original quantitative lipidomics data matrix, containing missing values where lipid species were undetected
2. mouse_lipidomics_data_transformed_imputed.csv: the same data structure with all missing values imputed
3. mouse_lipidomics_metadata.csv: sample-level metadata for all 26 samples

The following paragraphs describe the structure and content of each file in detail.

1. mouse_lipidomics_data_raw.csv

This file contains the original, unprocessed quantitative lipidomics data matrix [1]. It comprises 26 rows, each representing a unique biological sample collected from one of four tissue types: plasma (PL), cerebellum (CEREB), hippocampus (HPC), or prefrontal cortex (PFC).

The first column (“ID”) lists arbitrary subject identifiers; however, per treatment the order of samples corresponds to the order of study inclusion.

The next 62 columns correspond to individual lipid species measured in each sample from each tissue type, covering a range of lipid classes such as endocannabinoids, ceramides, sphingoid bases, and lysophosphatidic acids (Table 1). Specifically, the 62 quantitative lipid profiles (ng/ml) include lysophosphatidic acids (LPA: 16:0, 18:0, 18:1, 18:2, 18:3, 20:4), ceramides (C16, C18, C20, C24, C24: 1), sphingolipids (C16 sphinganine, C18 sphinganine, C24 sphinganine, C24:1 sphinganine, sphingosine, sphinganine, sphingosine-1P, sphinganine-1P), and endocannabinoids (eCBs: anandamide (N-arachidonoylethanolamine; AEA), palmitoylethanolamide (PEA), 1-arachidonoyl-glycerol (1-AG), 2-arachidonoylglycerol (2-AG), oleoyl-ethanolamide (OEA)), assayed using targeted liquid chromatography-tandem mass spectrometry (LC-MS/MS) methods described elsewhere [6]. Lipid species are denoted using a standardized nomenclature, with a tissue prefix and lipid abbreviation (e.g., “PFC_AEA” for anandamide in prefrontal cortex). The data are direct outputs from the mass spectrometry-based lipidomics workflow. Notably, the matrix contains 85 missing values (NaNs), which reflect undetectable or unquantifiable lipid species in specific samples.

2. mouse_lipidomics_data_transformed_imputed.csv

This file contains the processed lipidomics data matrix for the same 26 samples and 62 lipid variables as the raw dataset, but log_10_-transformed and with all missing values imputed. The first column again contains the arbitrary subject IDs, followed by the 62 lipid variables.

**Fig. 1:**
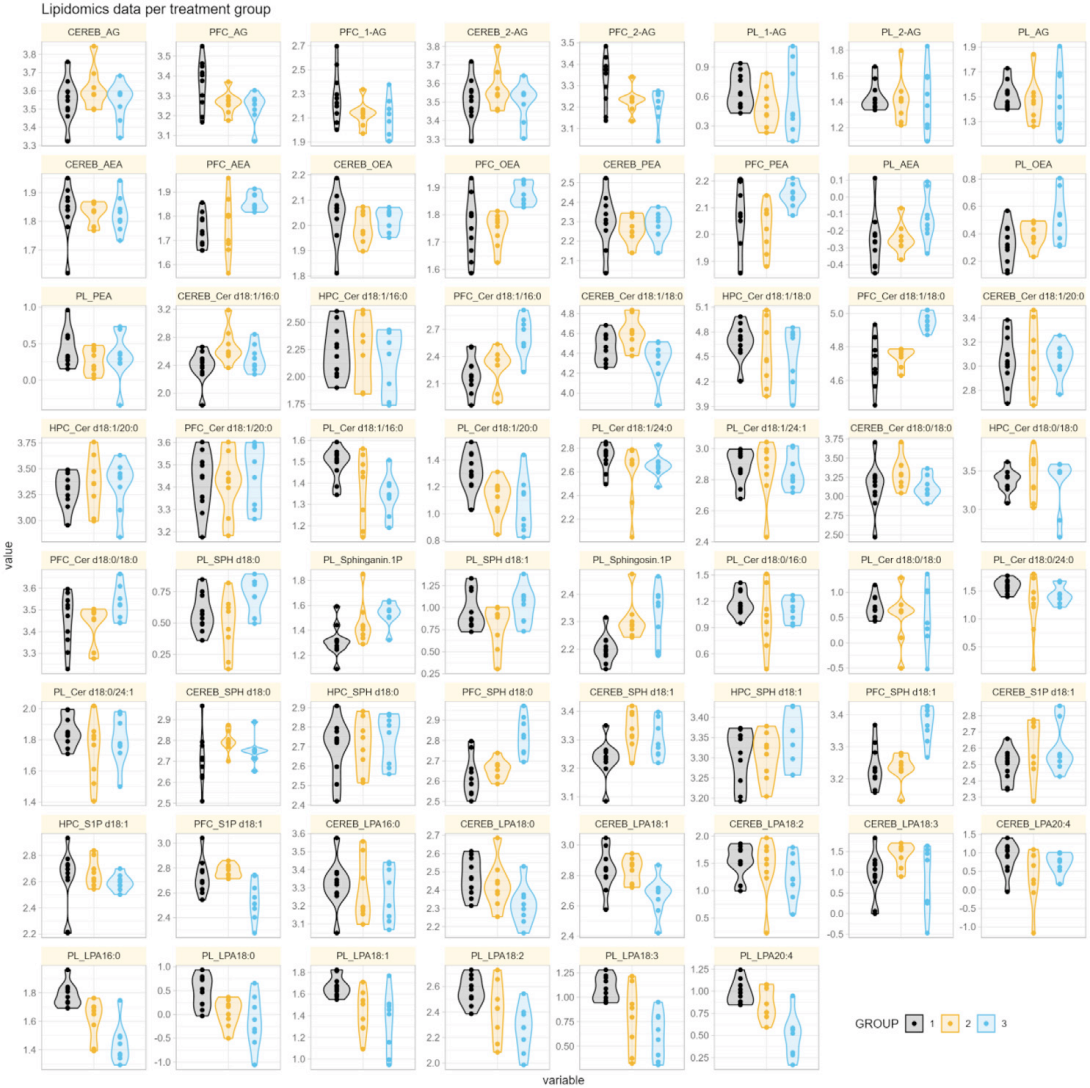
Quantitative lipidomics profiles of 62 lipid variables across cerebellum (CEREB), hippocampus (HPC), prefrontal cortex (PFC), and plasma (PL) tissues. Lipid abundances were measured by mass spectrometry in 26 mice, with variables labeled as tissue prefix plus standardized lipid abbreviation (e.g., “HPC_Cer d18:1/16:0” for ceramide d18:1/16:0 in hippocampus, “PL_LPA18:1” for lysophosphatidic acid 18:1 in plasma, or “PFC_AEA” for anandamide in prefrontal cortex). All variable names in the figure correspond exactly to the processed dataset column names and follow established lipidomics nomenclature, using the full chemical designation (e.g., “Cer d18:1/16:0” for ceramide; “SPH d18:0” and “S1P d18:1” for sphingoid bases and sphingosine-1-phosphate; “LPA18:1” for lysophosphatidic acid). Missing values were imputed prior to analysis, and data are presented for three experimental groups as defined in the metadata. Values indicate the relative or absolute abundance of each lipid species. Abbreviations: CEREB, cerebellum; HPC, hippocampus; PFC, prefrontal cortex; PL, plasma; Cer, ceramide; SPH, sphingoid base; S1P, sphingosine-1-phosphate; AEA, anandamide; OEA, oleoylethanolamide; PEA, palmitoylethanolamide; AG, monoacylglycerol.

The original dataset contained 5.3 % missing values (85 out of 1612 data points), which were addressed using a systematic evaluation of imputation strategies as previously described [7]. Both univariate (mean, median, mode) and multivariate methods were compared, including regression tree bagging as implemented in the R package “caret” [8] and random forest imputation using the “missForest” R package [9,10]. Method performance was assessed by artificially increasing the missing value rate and calculating the root mean squared percentage error (RMSPE) [11] between imputed and true values. Random forest imputation was selected as the final method based on its lowest median RMSPE and robust performance in this dataset. This results in a complete data matrix suitable for statistical or machine learning-based analyses. Fig. 1 shows a graphical overview of the distribution of the lipid variables, separately for the three treatment groups.

3. mouse_lipidomics_metadata.csv

The metadata file provides detailed contextual information for each of the 26 samples from the lipidomics datasets. Each row corresponds to a sample and includes four columns capturing sample identifiers and experimental group assignments.

The first column enumerates subject IDs that are concordant with the other files. Columns 2 to 4 delineate three classification variants:

• “DRUG”: The study examined the role of fingolimod medication, with the following coding system: 0 = placebo, 1 = fingolimod.
• “EAE”: The EAE model as an experimental model of multiple sclerosis was induced (coded with 1) or not (coded with 0).
• “GROUP”: The three study groups result from the former classification criteria as follows: 1 = no experimental autoimmune encephalomyelitis (EAE) and no drug serving as control (*n* = 10), 2 = EAE model (*n* = 8), and 3 = EAE plus fingolimod (*n* = 8).

This metadata enables the integration of lipid abundance data with experimental conditions, facilitating stratified analyses by tissue, group, or other covariates. All three files are aligned by sample order, ensuring straightforward cross-referencing between lipid abundance measurements and sample metadata.

## 4 Experimental Design, Materials and Methods

The dataset was generated from a preclinical investigation of lipid signaling mediators in the relapsing-remitting experimental autoimmune encephalomyelitis (EAE) mouse model, as previously described [1,2]. The study was conducted on 26 eight-week-old female SJL/J mice (Charles River, Sulzfeld, Germany) housed under standard laboratory conditions (12-hour light/dark cycle, 20–22 °C, 30–40 % humidity) with ad libitum access to food and water. All animal procedures were approved by the local Ethics Committee for Animal Research (Darmstadt, Germany) and were performed in accordance with institutional and national guidelines.

EAE was induced by subcutaneously injecting the PLP139–151/CFA emulsion with pertussis toxin (Hooke Kit™ PLP139–151/CFA Emulsion with PTX, EK-2120) according to the manufacturer’s protocol. Control animals received the corresponding antigen-free emulsion (Hooke Control Kit™, CK-2120). The mice were randomly assigned to three groups: (i) the control group (no EAE; *n* = 10), (ii) the EAE group with vehicle treatment (*n* = 8), and (iii) the EAE group with fingolimod treatment (FTY720; *n* = 8). Fingolimod (Cayman Chemical, 98 % purity) was administered in the drinking water at a dose of 0.5 mg/kg/day starting 18 days after immunization. The vehicle formulation contained 60 % tap water, 39.96 % PBS, and 0.04 % HCl (37 %).

At the experimental endpoint, plasma and brain tissue samples (from the cerebellum, hippocampus, and prefrontal cortex) were collected for lipidomics analysis. We performed lipid extraction and quantification of 62 lipid variables of four species using targeted liquid chromatography-tandem mass spectrometry (LC-MS/MS), as previously described [6]. Briefly, samples were handled under standardized conditions: blood was collected into *K*+EDTA tubes, rapidly processed to separate plasma, snap-frozen, and stored at −70 °C to minimize pre-analytical variability. Depending on lipid class, specific liquid–liquid extraction (LLE) procedures were applied for extraction: ethyl acetate:hexane (9:1, v/v) was used for endocannabinoids and related lipids; a chloroform:methanol:hydrochloric acid mixture was used for sphingolipids and ceramides; and butanol extraction was used for lysophosphatidic acids. Isotopically labeled internal standards were added to all samples to ensure accurate quantification.

Quantitative analysis of lysophosphatidic acids (LPAs) was performed using liquid chromatography-electrospray ionization-tandem mass spectrometry (LC-ESI-MS/MS) on a hybrid triple quadrupole-ion trap QTrap 5500 mass spectrometer (AB Sciex, Germany), as previously described [1,12,13]. Briefly, LPAs were extracted from 50 µL samples via two cycles of liquid-liquid extraction with 1-butanol after the addition of 20 µL of the internal standard LPA 17:0. The combined organic phases were evaporated under nitrogen at 45 °C and reconstituted in 200 µL of methanol. Chromatographic separation was achieved using a Mercury C18 column (20 × 2 mm, 3 µm, 100 Å) with a precolumn and a linear gradient at a flow rate of 400 µL/min. The mobile phases consisted of water containing 50 mM ammonium formate and 0.2 % formic acid (phase A) and acetonitrile containing 0.1 % formic acid (phase B). Negative ion multiple reaction monitoring (MRM) was used for detection. Quantification was based on the internal standard method using Analyst Software V1.5 (Applied Biosystems, Germany). Calibration curves were linear over the range of 0.1–500 ng/mL with an accuracy exceeding 95 %. All analyses were performed blinded to treatment groups.

## Limitations

The sample size (*n* = 26) was determined based on statistical recommendations from experts to ensure sufficient power for the experimental design. However, as with many targeted preclinical studies, the dataset remains limited to a single strain and sex (female SJL/J mice) and specific experimental conditions, which may affect generalizability to other populations, strains, or sexes. The presence of missing values (5.3 %) due to undetectable or low-abundance lipid species was addressed through the implementation of validated imputation methods. However, it is important to note that imputed values may not fully capture the underlying biological variability. The quantification of lipids was conducted at a single temporal point, thereby constraining temporal resolution. It should be noted that technical replicates were not included in the study. When considering reuse or extension of the dataset, these factors should be considered.

## Ethics Statement

These experiments adhered to the guidelines of the Committee on Research and Ethical Issues of the International Association for the Study of Pain (IASP), as well as the GV-SOLAS guidelines for animal welfare in science and the ARRIVE guidelines. The experiments were approved by the local Ethics Committee for Animal Research in Darmstadt, Germany (ID F95-46 and F152-02).

## CRediT authorship contribution statement

**Jörn Lötsch:** Software, Validation, Data curation, Writing – original draft, Writing – review & editing, Visualization, Funding acquisition. **Irmgard Tegder:** Validation, Formal analysis, Investigation, Data curation, Writing – review & editing, Supervision. **Natasja de Bruin:** Methodology, Investigation, Data curation, Writing – review & editing. **Dominique Thomas:** Formal analysis, Investigation, Resources, Data curation, Writing – review & editing. **Gerd Geisslinger:** Writing – review & editing.

## Data Availability

Mendeley DataTargeted lipidomics dataset of central nervous system and plasma from mice with experimental autoimmune encephalomyelitis (Original data).
